# 21746 Antigen discovery in membranous glomerulopathy using laser capture microdissection and mass spectrometry

**DOI:** 10.1017/cts.2021.405

**Published:** 2021-03-30

**Authors:** Jacquelyn Fede, Stephen Kogut, Anthony Heyward, John F. Stevenson, Amy Nunn, Julie Plaut, Judy A. Kimberly

**Affiliations:** 1 University of Rhode Island; 2 Warren Alpert Medical School, Brown University; 3 Brown University

## Abstract

ABSTRACT IMPACT: Identifying the causative antigen in membranous glomerulopathy cohorts enables the development of serum assays to detect and monitor disease progression without the need for invasive kidney biopsies. OBJECTIVES/GOALS: Primary membranous glomerulopathy is caused by the formation of autoantibody immune complexes which deposit in the glomerulus and obstruct kidney function. Causative antigens remain to be identified in roughly 20% of cases. Our goal is to identify the antigen in these cohorts, so that non-invasive assays can be developed for disease monitoring. METHODS/STUDY POPULATION: Renal biopsy tissue from known antigen cases (PLA2R, THSD7A), and unknown cases were included in the analysis. Renal biopsy tissue from formalin fixed paraffin embedded tissue was cut at a thickness of 10 µm onto Leica PET-membrane frame slides. These slides were then stained with hematoxylin. The glomeruli were microdissected into microcentrifuge tubes using a Leica DM6000B microscope. The microdissected glomeruli were lysed in 2% SDS and 0.1M DTT at 99 degrees Celsius for 1 hour and processed by filter assisted sample preparation (FASP). Digested peptides were analyzed by liquid chromatography-mass spectrometry using an Orbitrap Fusion Lumos using data-dependent acquisition. RESULTS/ANTICIPATED RESULTS: Mass spectrometry data collected from the laser captured glomeruli was searched against the human proteome fasta database from Uniprot using MaxQuant. IBAQ values were used for quantitation and statistical analysis. Null hypothesis significance testing was performed for each protein by comparing each sample group to the rest of the samples in the data set. In the control groups, the causative antigens PLA2R and THSD7A were detected and quantified with the largest magnitude fold change in their respective category, validating the experimental design. Using this approach, the proteins SAP, NELL1, and NCAM1 were identified and subsequently validated as causative antigens in distinct patient cohorts. DISCUSSION/SIGNIFICANCE OF FINDINGS: Here, we share the results of our efforts to comprehensively identify the spectrum of causative antigens in membranous glomerulopathy. In this context, antigen discovery is an essential first step for the development of non-invasive assays to inform prognosis, monitor response to treatment, and better understand disease etiology.

